# Transversus Abdominis Muscle Release in Giant Incisional Hernia

**DOI:** 10.7759/cureus.28277

**Published:** 2022-08-22

**Authors:** Stanko Baco, Milos Mitric

**Affiliations:** 1 General Surgery, Public Health Institution Hospital “Dr Mladen Stojanović”, Prijedor, BIH

**Keywords:** mesh repair, complex abdominal wall reconstruction, giant incisional hernia, anterior components separation, posterior components separation, transversus abdominis release

## Abstract

Giant incisional hernias and complex abdominal wall reconstructions are challenging in terms of primary closure with typical operating techniques. Transversus abdominis muscle release (TAR) is a new myofascial release technique that involves the creation of a retro rectal place and mesh placement. It is a modification of the posterior component separation technique (CST) and enables the primary closure of the most challenging abdominal wall reconstructions. We present a case where a favorable outcome was achieved in a contaminated environment with almost no perioperative complications and no recurrence after 17 months, with the placement of inexpensive non-absorbable ‘Paha’ mesh.

## Introduction

Incisional ventral hernias are common complications of abdominal surgery. Giant ventral hernias and complex abdominal wall reconstructions, on the other hand, are challenging tasks even for the most experienced hernia surgeons with no consensus as to which technique is the best suited for these procedures. The Rives-Stoppa-Wantz technique is a proven technique for ventral hernia repairs, but because of the limitation related to the rectus sheath, it is not a viable option for giant hernias and wall defects [1]. Unfavorable outcomes with traditional sutures and mesh techniques have led to the development of numerous new techniques. The classic (open anterior) component separation technique (ACST) was first reported by Albanese and later popularized by Ramirez as an autologous tissue repair technique [2,3]. However, its association with high recurrence rates has resulted in new modifications. Posterior component separation (PCS) with the transversus abdominis muscle release (TAR) represents an evolution of the Rives-Stoppa-Wantz technique and presents a major advancement in the complex abdominal wall reconstruction [4,5].

## Case presentation

**Figure 1:**
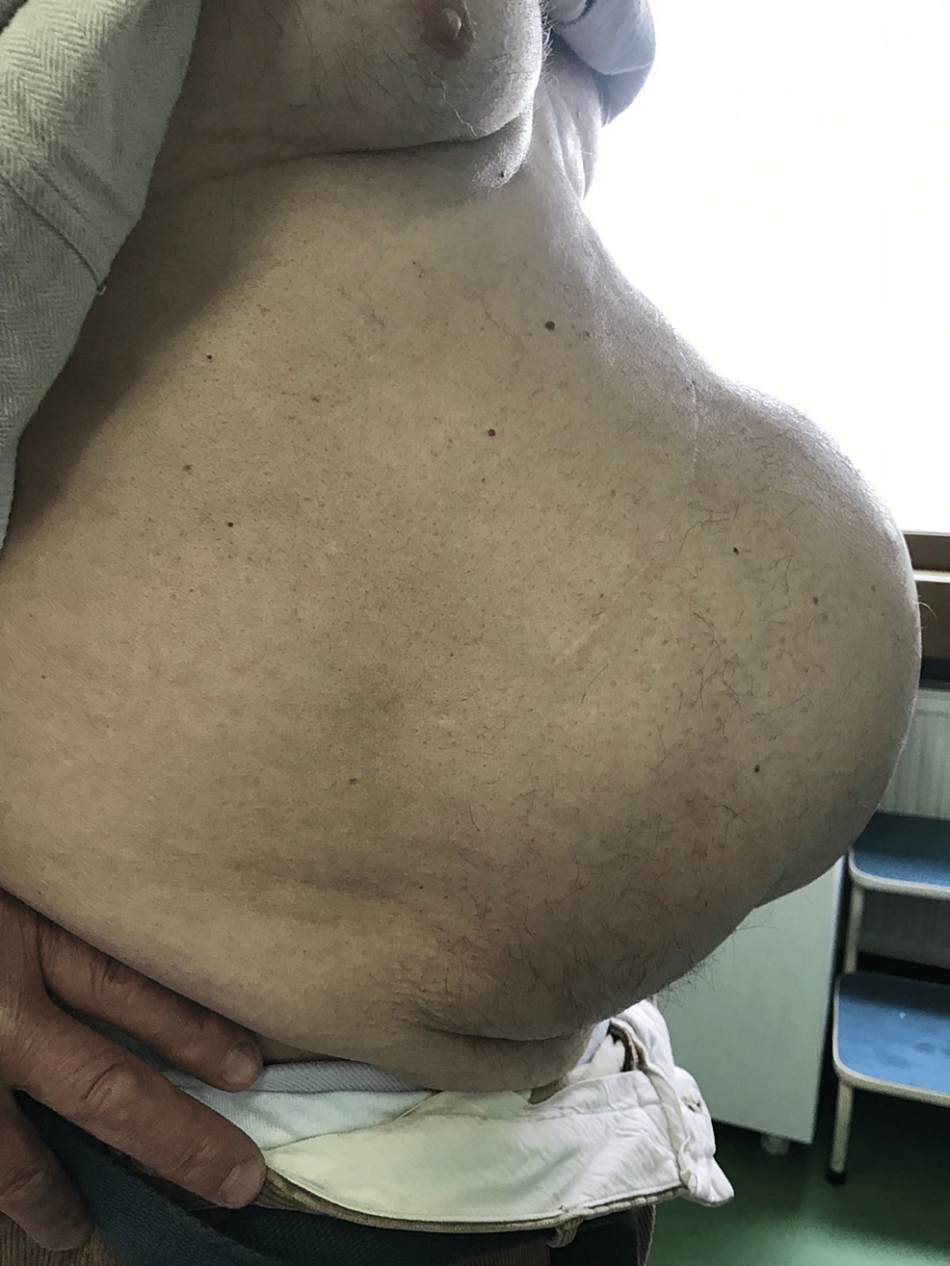
Preoperative lateral view of abdomen showing a giant ventral incisional hernia, developed after two abdominal operations

**Figure 2:**
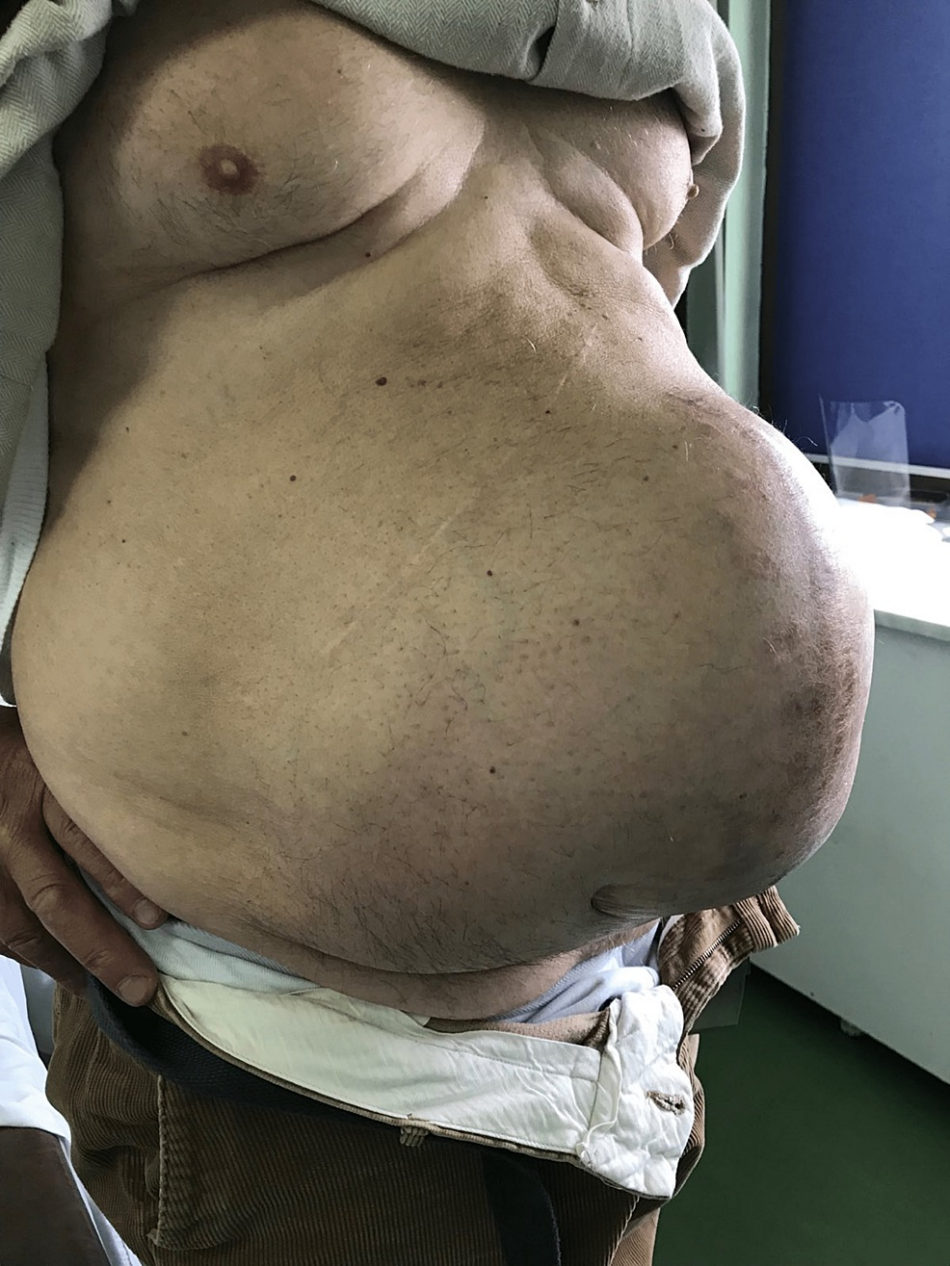
Preoperative oblique view of abdomen showing a giant ventral incisional hernia, developed after two abdominal operations

**Figure 3:**
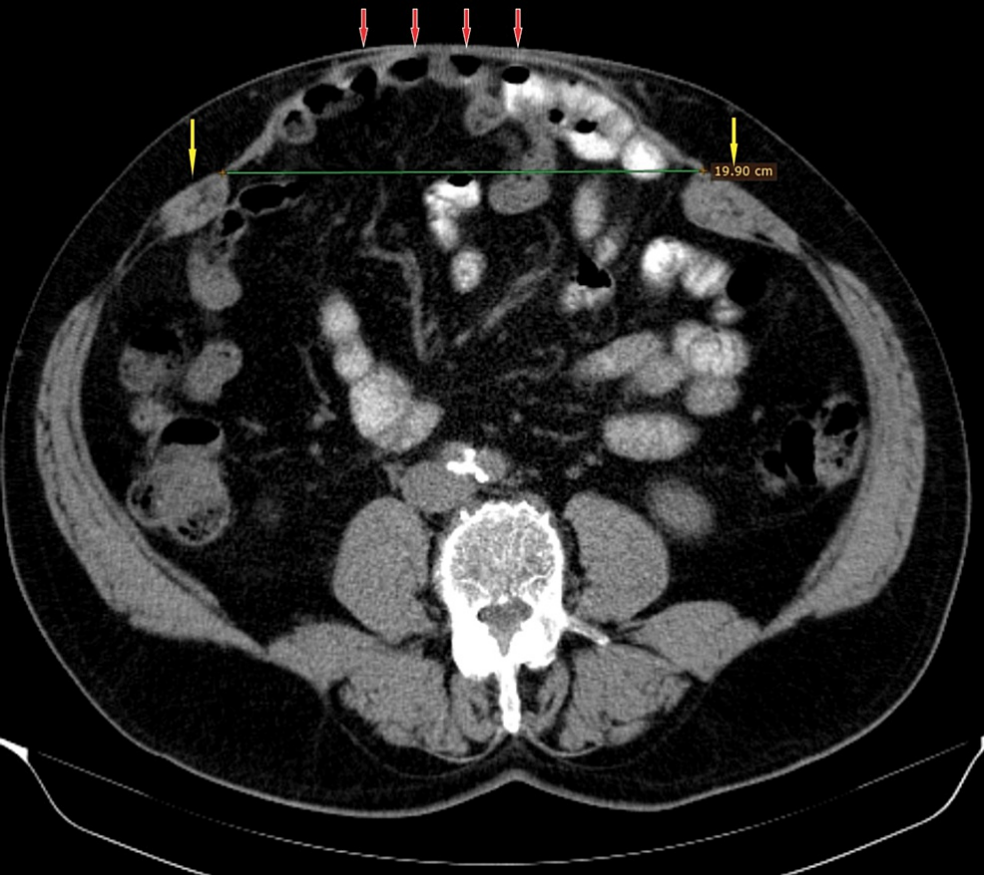
Preoperative abdomen and pelvis CT scan The image shows a giant incisional ventral hernia with a midline defect, maximum 19.9 cm in width, wide displacement and atrophy of the rectus abdominis muscle (yellow arrows), and herniated bowel loops adherent to the skin (red arrows) CT: computed tomography

**Figure 4:**
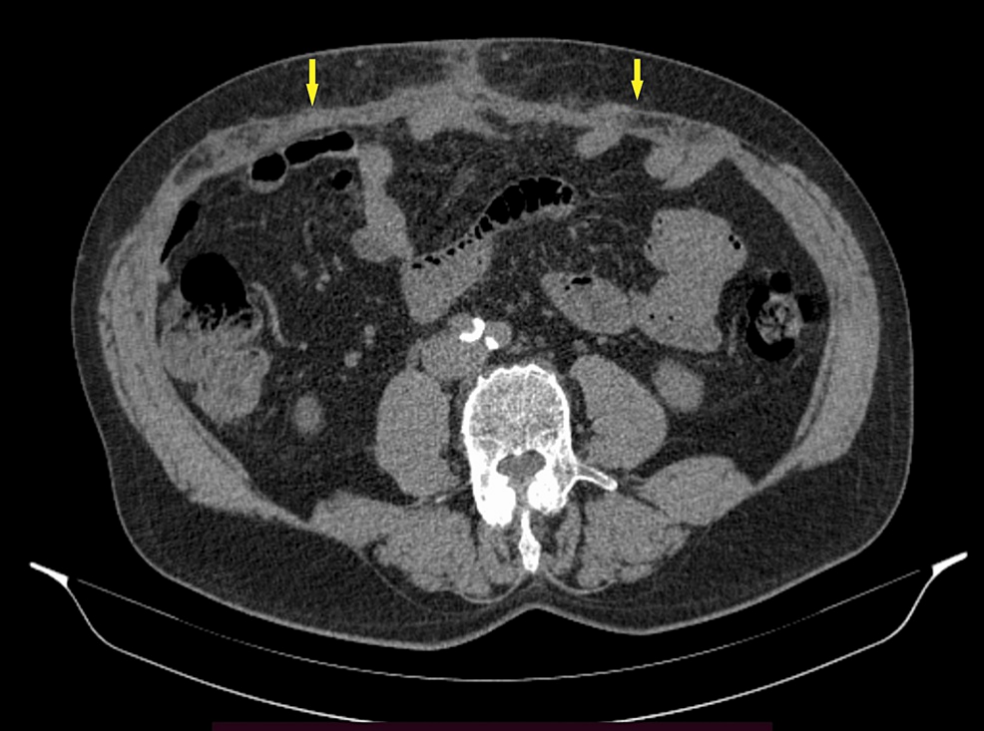
Postoperative control abdomen CT scan, 17 months after TAR, with restored abdominal wall continuity (arrows) CT: computed tomography; TAR: transversus abdominis muscle release

**Figure 5:**
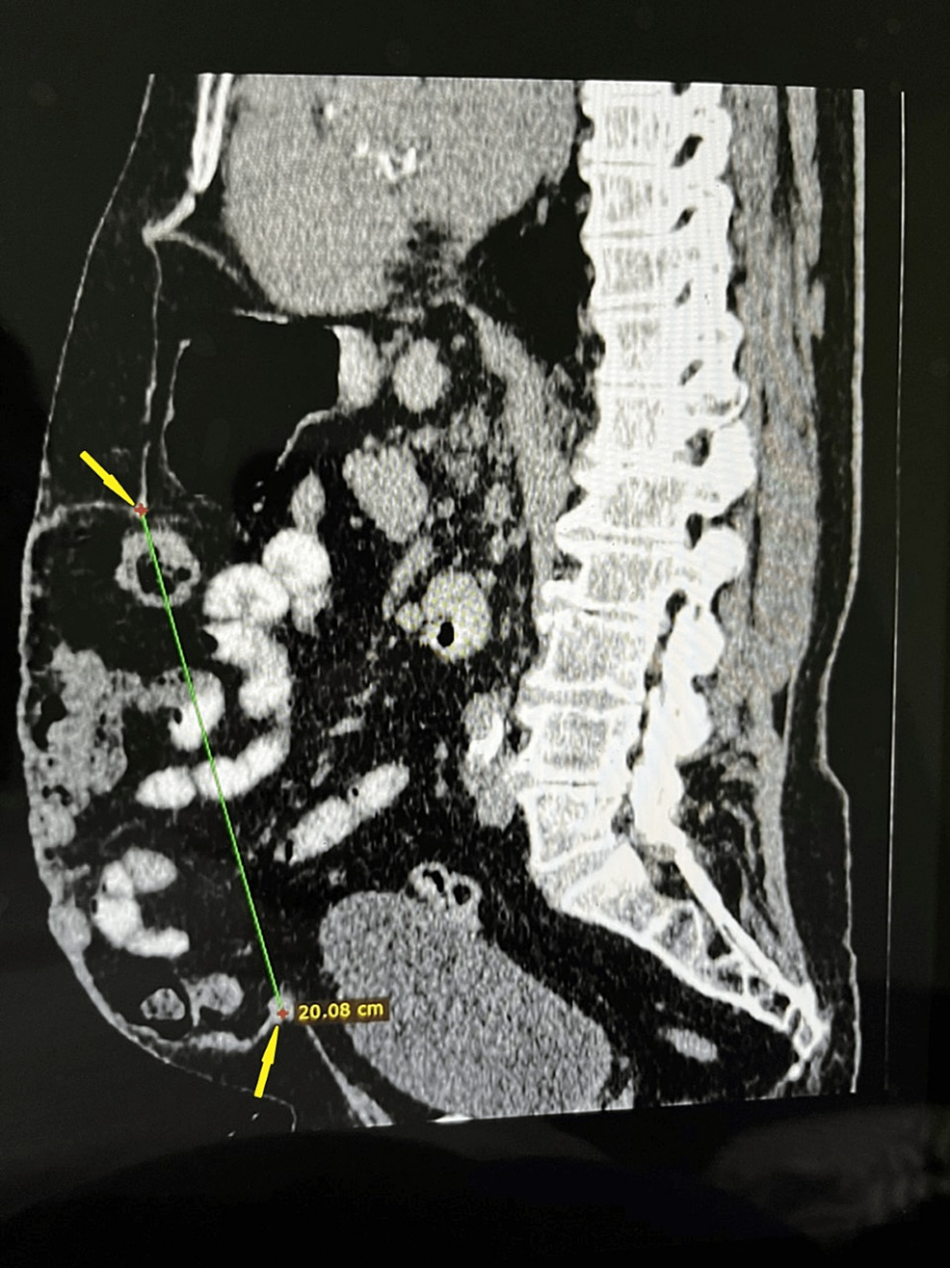
Preoperative sagittal abdomen CT scan with a 20-cm long midline defect CT: computed tomography

**Figure 6:**
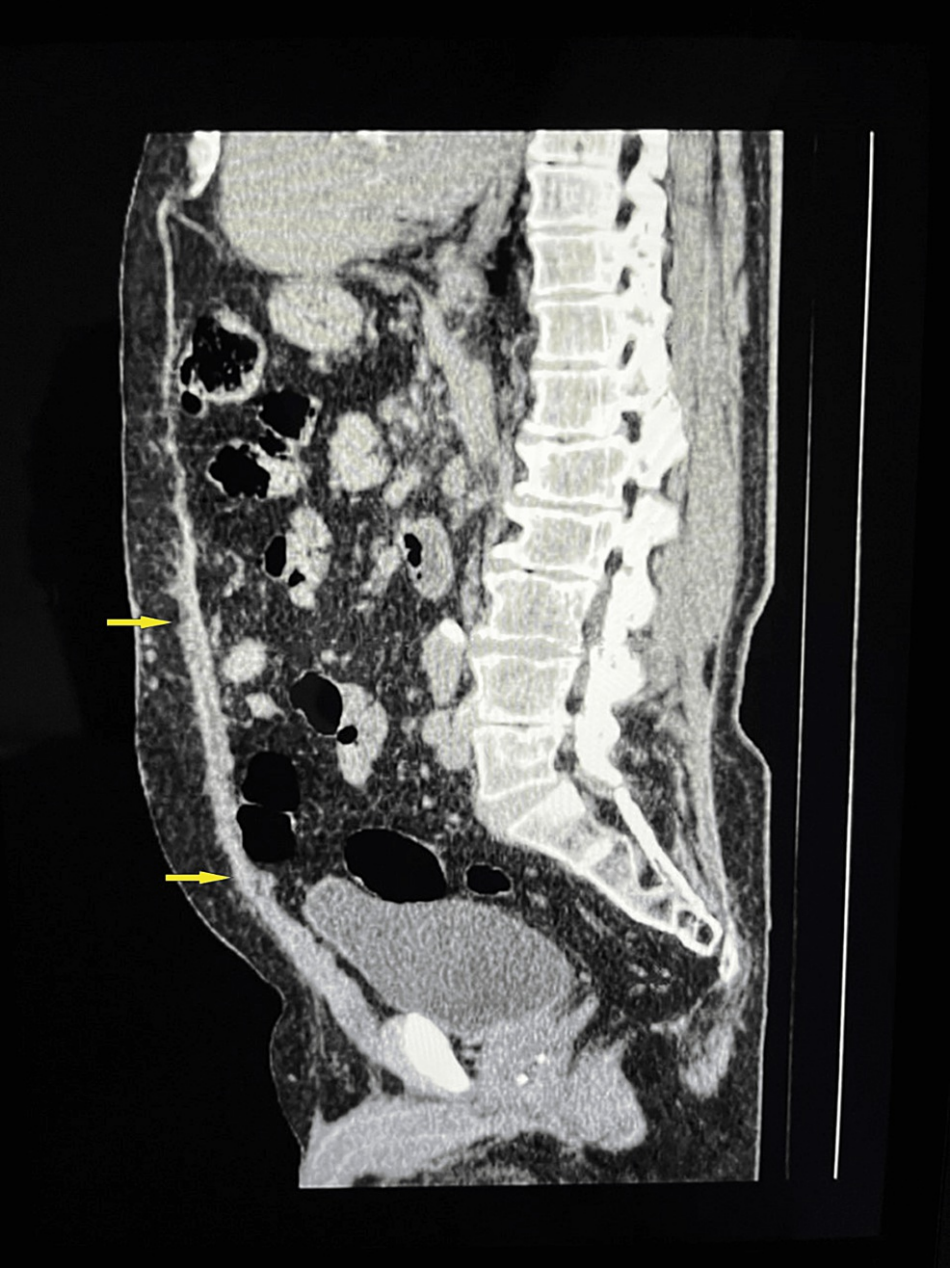
Postoperative sagittal control abdomen and pelvis CT scan, 17 months after TAR with restored abdominal wall continuity (yellow arrows) CT: computed tomography; TAR: transversus abdominis muscle release

A 70-year-old man, who had been twice operated on in our hospital, presented with a giant ventral incisional hernia for an elective operation. He had undergone an emergency laparotomy for the first time due to an adhesional ileus, with adhesiolysis of the small intestine and colonis sygmoidei, in April 2020. The postoperative course had been complicated by a medial laparotomy wound dehiscence and evisceration of the small intestine on the 12th postoperative day. After the reoperation with wound revision and closure, a surgical site infection developed, which led to the development of a giant incisional hernia. Over time, the hernia had increased in size without incarceration. At the time of examination, his general condition was good. Regarding comorbidities, he had experienced a chronic hepatitis B infection of about 80 x 40 cm from the anterior abdominal wall (Figures 1-2). The rectus defect in the umbilical region in the transverse direction was 19.9 cm wide and 20 cm in the vertical direction. The bowel could be palpated and the borborygmus heard under normally colored skin. CT of the abdomen showed a complete defect of the anterior abdominal wall with a defect area of 398 cm^2^ and the bowel adherent to the skin (Figures 3-6).

**Figure 7:**
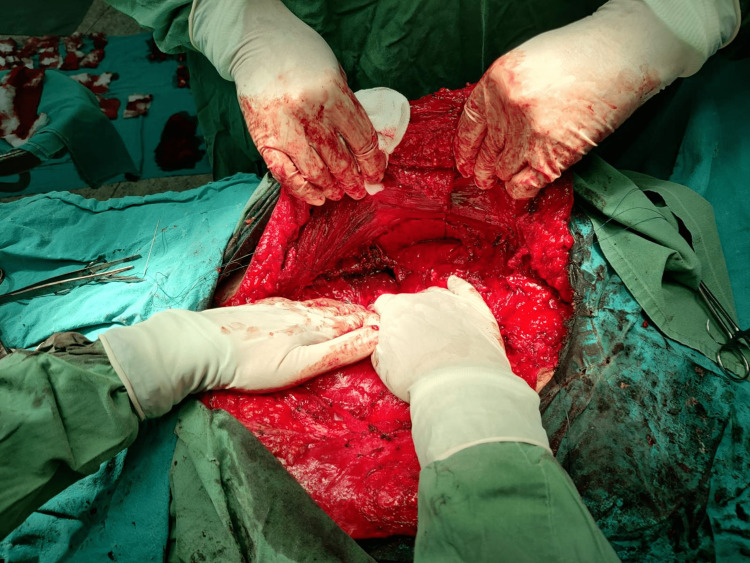
Transversus abdominis release: blunt separation of the fascia transversalis from transversus abdominis muscle

Surgery was planned at 11 months after the last operation. Preoperative bowel preparation and enemas had not been administered. The patient had taken only liquid food for two days before the operation. Deep vein thrombosis prophylaxis had been started from the day of admission. Perioperatively, the patient received intravenous only 1 g of cefazolin and 0.5 g metronidazole, covering the aerobic and anaerobic bacteria. The patient was obese with a BMI of 27.1 kg/m^2^. The abdomen was opened by a medial incision. On exploration, we did not find ascites or neoplastic processes. There were extensive interintestinal adhesions as well as adherence of the small bowel to the skin, and hence the unexpected need for a concurrent bowel surgery occurred. Due to the local findings, we were compelled to resect approximately 15 cm of the intestine tenui and constructed a T-T ileo-ileal anastomosis, alert to the risk of the operating field contamination. The rectus abdominis muscle was completely retracted laterally with a defect of almost 20 cm on the umbilicus level. The posterior rectus sheath (PRS) was incised on the medial border, close to the linea alba, and retro rectal dissection laterally to the linea semilunaris followed. The neurovascular bundles were preserved and the PRS incised as laterally as possible. The transversus abdominis muscle was visualized and divided with electrocautery, from 4-5-cm cranial to the xiphoid (diaphragm central tendon), laterally to the psoas muscle and caudally down to the retropubic Retzius space and approximately 5 cm caudal to Cooper's ligament (Figure 7).

**Figure 8:**
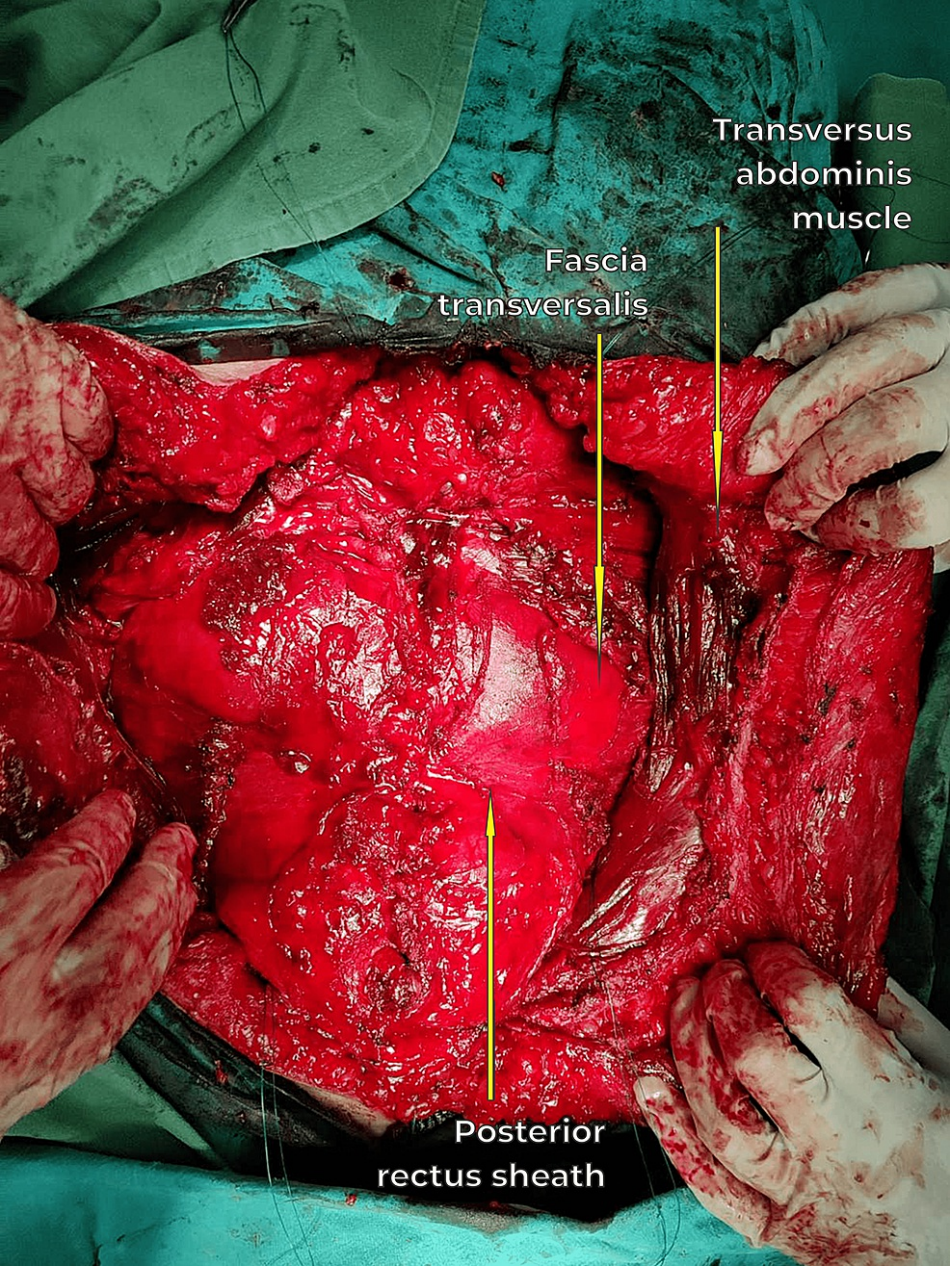
Xipho-pubic laparotomy, tears and holes of the peritoneum, and fascia transversalis closed with absorbable 2-0 sutures and retracted myofascial flaps

**Figure 9:**
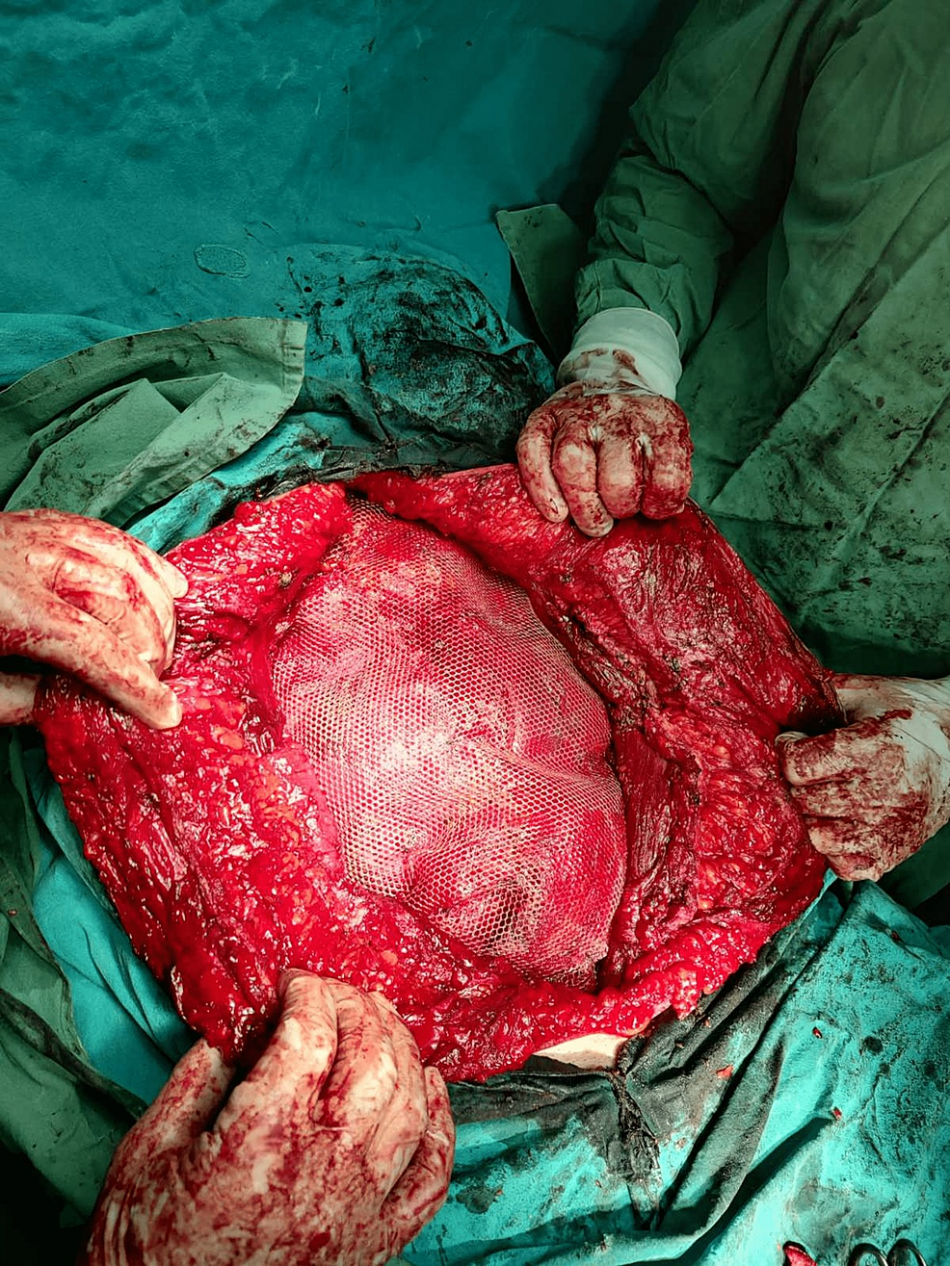
Two polypropylene mesh sized 30 x 30 cm in retro muscular (sublay) position and fixated with nylon 3-0

**Figure 10:**
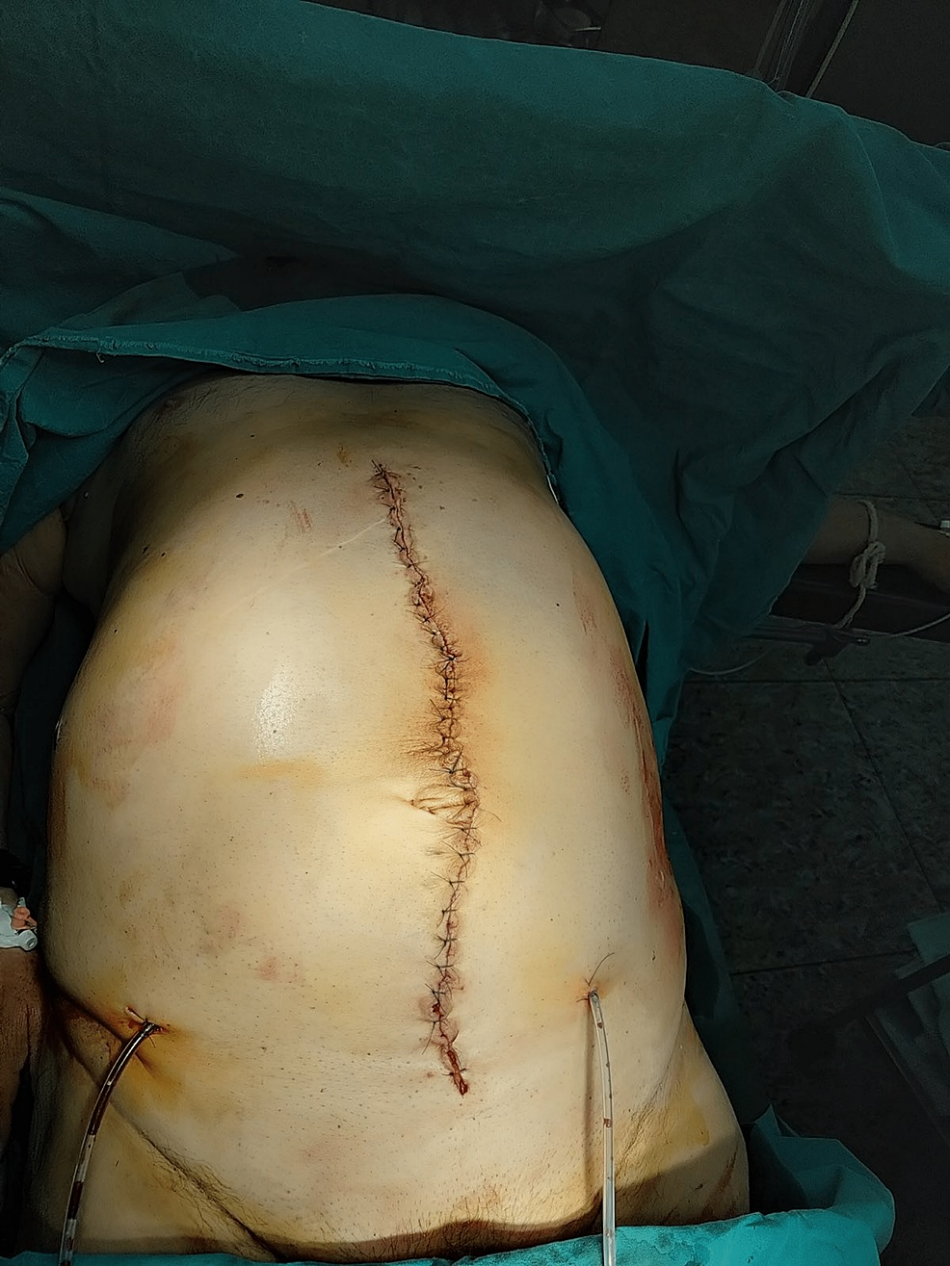
Immediate postoperative image

**Figure 11:**
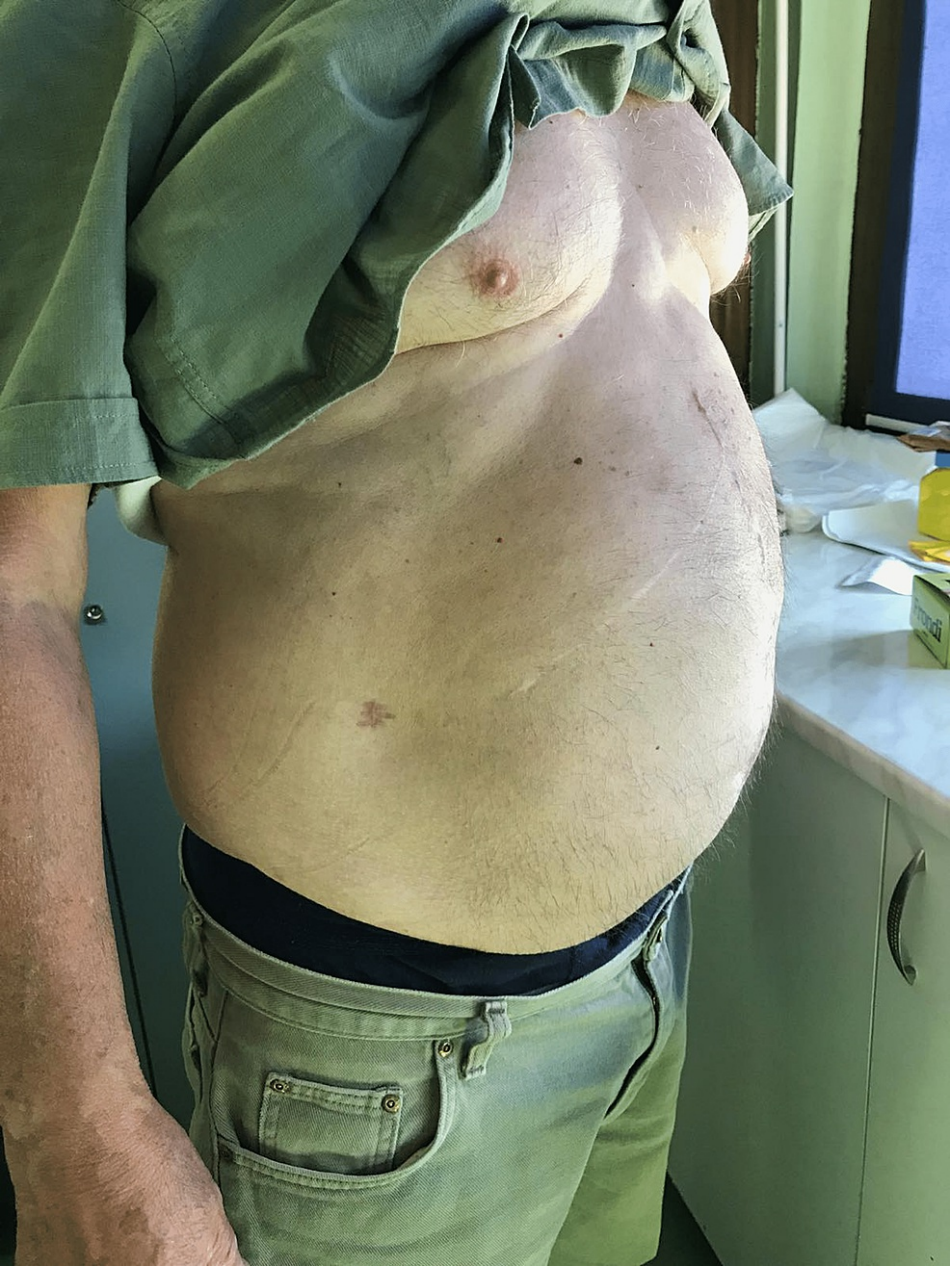
Six weeks after the operation

Unfortunately, due to prior operations, despite our efforts, there was not much of omentum to be interposed. The peritoneum and the fascia transversalis were approximated in the midline using absorbable Vicryl 2/0 sutures (some peritoneum fenestrations were repaired) (Figure 8). Two non-absorbable, monofilament polypropylene mesh sized 30 x 30 cm were placed retro muscular (sublay) and fixated with nylon 3/0 (Figure 9). The anterior rectus sheath could not be approximated. Two Jackson-Pratt closed suction drains were placed preperitoneal and the subcutaneous drains were omitted. After panniculectomy and scar excision, the skin was closed in the midline (Figure 10). The patient was shifted after two hours to the general surgery department with intravenous analgesia. Clear liquids were given on the first postoperative day. The bladder catheter was removed on the fourth postoperative day and the drains on the 10th day, after a significant decrease in output. The patient was discharged on the 11th day and advised to wear the binder all the time, except while sleeping. A minor postoperative complication was the development of a wound seroma, which spontaneously resolved through a wound opening in the periumbilical region, sized approximately 2 cm. The sutures were removed after three weeks (Figure 11).

## Discussion

Ventral incisional hernia repair is one of the most commonly performed general surgical operations. The Rives-Stoppa-Wantz hernia repair technique, described in 1973, with the retro rectus abdominis muscle mesh placement, much larger than the hernial defect, is a proven and effective technique for open ventral hernia repairs. The major disadvantage of the technique is the limitation to the rectus compartment. Sometimes, in cases of giant ventral hernias with extensive tissue loss or even loss of domain, the Rives-Stoppa-Wantz technique is not sufficient to achieve a mesh overlap, a tension-free midline approximation, and restoration of the normal anatomy of the abdominal wall, and hence the recurrence risk is very high. The concept of CST was developed due to the unfavorable results of traditional techniques without the relaxing of the musculofascial flaps. The classic open anterior CST was first reported by Albanese and popularized by Ramirez decades later. It involves the functional reconstruction of the abdominal wall with autologous tissue repair. Wide skin flaps are created to access the external oblique aponeurosis. The external oblique aponeurosis is divided longitudinally lateral to the rectus compartment and separated from the underlying internal oblique muscle. The myofascial flap consisting of the rectus, internal oblique, and transversus abdominis muscle (TAM) is mobilized medially [2-6]. Because of high complication rates, many new techniques have been developed [7].

Milburn et al. (2007, cadaver trial) and Carbonell et al. (2008) [4] reported a posterior CST (PCST) technique. It involves the incision of the PRS, after the separation from the rectus muscle, at its most lateral edge with access to the plane between the internal oblique and TAM. The fact that the plane between the internal oblique and TAM contains nerves and blood vessels leads to the main disadvantage of this technique - cutting of the intercostal nerves while advancing from the rectus abdominis to the lateral compartment, which may result in partial or complete rectus muscle paralysis [5,6,8,9].

The problem of extending the dissection from the rectus abdominis muscle to the lateral compartment was resolved by Novitsky et al., who reported TAR as a new technique: a modification of the PCST and an extension of the Rives-Stoppa-Wantz [7-9]. It represents one of the major advances in abdominal wall surgery in the last decades and is indicated for very large hernias or combined abdominal wall defects. Mesh is placed sublay; retro muscular and perforators to the rectus muscle are identified and preserved (longitudinal incision of the PRS 1.5-2 cm medial to the lateral border of the rectus compartment) [9]. It offers superior results over non-mesh reinforced repairs [10-11]. TAR could be an ideal hernia repair technique for complex defects because it weakens the lateral sides of the abdominal wall to augment the midline repair, thereby also increasing the lateral abdominal wall compliance and reducing the pressure on the midline [10-13]. However, TAR should not be combined with anterior CST.

## Conclusions

Our case shows that even the most challenging abdominal wall hernias can be reconstructed with TAR. It is a safe, effective, and reliable technique with low perioperative morbidity as well as reduced risk of skin necrosis and surgical site infection. Recurrence rates, however, are yet to be analyzed. Additionally, the successful use of synthetic mesh reinforcement (in the most suitable retro muscular sublay plane), even in a contaminated environment, is possible, leading to a favorable outcome.
